# AI Approaches to Homogeneous Catalysis with Transition Metal Complexes

**DOI:** 10.1021/acscatal.5c01202

**Published:** 2025-05-14

**Authors:** Lucía Morán-González, Arron L. Burnage, Ainara Nova, David Balcells

**Affiliations:** † Hylleraas Centre for Quantum Molecular Sciences, Department of Chemistry, 6305 University of Oslo, P.O. Box 1033, Blindern, 0315 Oslo, Norway; ‡ Centre for Materials Science and Nanotechnology, Department of Chemistry, University of Oslo, 0315 Oslo, Norway

**Keywords:** Homogeneous Catalysis, Transition Metal Complexes, Artificial Intelligence, Machine Learning, Reaction Mechanism, Catalyst Discovery, Catalyst Design, Catalyst Optimization

## Abstract

Artificial intelligence (AI) is transforming research in chemistry, including homogeneous catalysis with transition metals. Over the past 15 years, the number of publications combining AI with catalysis has increased exponentially, reflecting the interest and strength of this strategy in the field. Since this is a broad emerging discipline, it is essential to establish guidelines that clarify the diverse approaches already available. The complexity of the tasks that can be carried out with AI tools is directly linked to the nature of their components, including datasets, representations, algorithms, and high-throughput experimental and computational facilities. In parallel to the evolution of these tools, applications to catalysis have also advanced. Initially, models were developed to predict key aspects of the reaction mechanism, aiming at screening catalyst candidates. Subsequent studies have incorporated experimental data to optimize reaction conditions and yields. More recently, generative AI based on deep learning methods has enabled the inverse design of novel catalysts with predefined target properties. While most studies rely on computational data, recent advancements have improved the acquisition of experimental data, enabling AI-driven automated workflows. This Perspective gives a critical overview on selected studies that reflect the state of the art in the application of AI to homogeneous metal-catalyzed reactions, also highlighting future opportunities and challenges.

## Graphical abstract

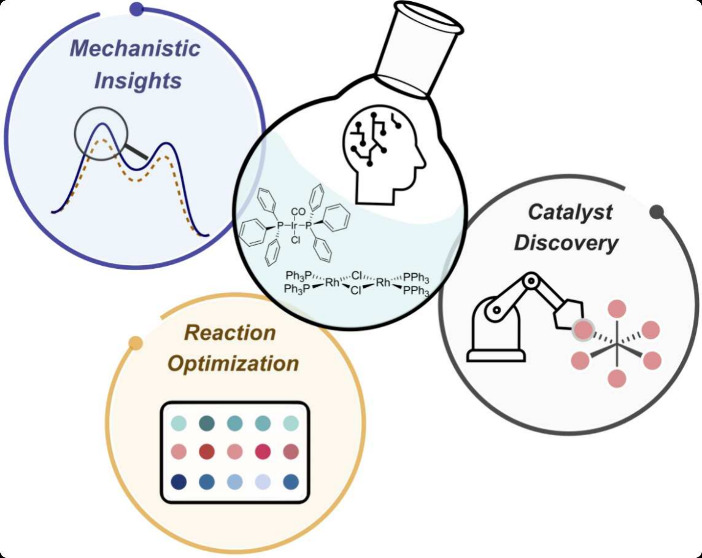

## Introduction

Catalysis plays a vital role in everyday life, from powering vehicles^1,2^ and manufacturing plastics^3,4^ to synthesizing life-saving pharmaceuticals.^5^ It is estimated that nearly 90% of all products we use today involve catalytic processes at some stage of their production.^6^ Nobel Laureate Benjamin List underlined the significance of catalysis in his lecture: “catalysis is among the most relevant cultural human accomplishments in the history of mankind.... It is probably the single most important technology for our future.”^7^ By harnessing catalytic processes, we accelerate chemical reactions, reduce costs, and enhance overall efficiency.^8–10^ Hence, this approach is pivotal for addressing environmental challenges, minimizing the generation of waste and the consumption of energy, addressing at least eight of the United Nations’ Sustainable Development Goals.^11^

Catalysis is commonly categorized into three main groups: homogeneous, heterogeneous,^12^ and enzymatic.^13,14^ As illustrated on the left-hand side of Figure 1, the number of publications over the last 15 years shows an intensive effort toward enzymatic catalysis, which can be related to the rising prominence of protein research in the Nobel Prizes in Chemistry.^15^ Heterogeneous and homogeneous catalysis represent 27% of the work published, with the latter showing a steady increase that consolidated in 2016. Catalysis has been also conceptualized from other angles, as reflected by the common use of alternative classifications based on energy source (for example, photocatalysis^16,17^ ), selectivity (for example, asymmetric catalysis^18,19^ ), or catalyst type (for example, organocatalysis^20^). Homogeneous catalysis constitutes a significant ∼15% of all catalytic processes^21^ and plays a major role in the industry of fine chemicals, including agrochemicals and drugs. Other important industrial processes are the Monsanto acetic acid production,^22^ hydroformylation,^23^ Wacker reaction,^24^ and C–E cross-coupling reactions^25–27^ (E = C or N). Homogeneous catalysis offers the advantage of referring to a well-defined molecular catalyst that can be designed at the atomic level to optimize not only activity, but also selectivity and robustness. However, it also has the drawback of being difficult and expensive to recycle.^28^

**Figure 1:**
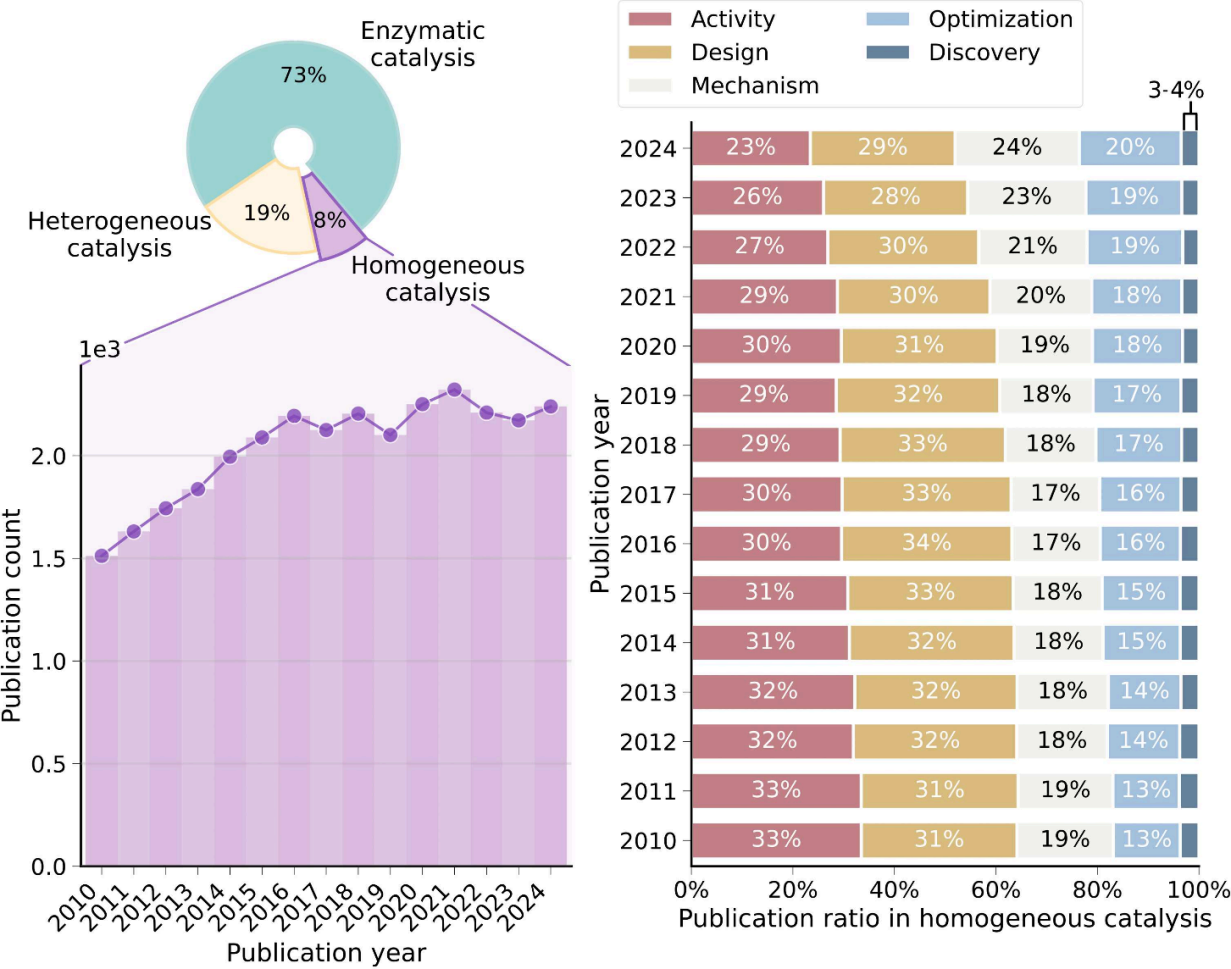
(left) Number of publications over time on the topic of homogeneous catalysis. The pie chart shows the proportion of publications in this area relative to heterogeneous and enzymatic catalysis. (right) Publication ratios on the topic of homogeneous catalysis over the keywords “activity”, “design”, “mechanism”, “optimization”, and “discovery” found in their titles or abstracts. All data in both panels are for the 2010–2024 time range. Source: Web of Science.

Transition metal complexes (TMCs) have historically attracted the interest of the homogeneous catalysis community due to their modular nature, in which the properties of the metal center promoting bond cleavage and formation, can be finely tuned over ample, and interrelated, electronic and steric scales, thanks to the large and diverse ligand libraries currently available. This generates vast chemical spaces, opening opportunities to solve problems of interest like, for example, the substitution of the prevalent noble metals by the earth-abundant 3d metals,^29–31^ or the design of novel chiral ligands for asymmetric synthesis.^32,33^ However, the efficient exploration of these spaces is hampered by the high cost of synthesizing, characterizing, and testing TMCs in catalytic processes. Brute-force screening should thus be done cleverly in a context in which intelligence, both human and artificial, is key.

Artificial intelligence (AI) is pushing a paradigm shift in many fields, including catalysis.^34–41^ This Perspective focuses on the application of AI to homogeneous catalysis with TMCs, including also a few examples on organocatalysis. The term AI is used instead of machine learning (ML), since it defines a broader framework comprising elements that are becoming increasingly important in chemistry, like robotic systems that make lab operations not only automated but also autonomous.^42–44^ This is a key capability enabled by AI that makes it distinct from traditional methods heavily based on heuristics and rules. In this regard, one can make an analogy with the process of using a cooking recipe. In a predetermined approach, a recipe would provide precisely defined ingredients in exact quantities, along with the detailed steps to follow in order to achieve the target dish, in a way that can be highly efficient but limits the learning of the cook. In the AI way, the ingredients, which can be seen as the data, are provided, but the recipe is flexible, allowing the cook to proceed autonomously in a way that can improve the final dish, influenced by knowledge on similar dishes as well as by past experience and external interventions that can occur during the cooking. Here, the cook should also be capable of defining the recipe of a finished dish shown to him.

The right-hand side of Figure 1 reflects the focus of homogeneous catalysis studies on different topics and how this has changed during the last 15 years. While the interest in catalyst activity and design has been diminishing and consolidating, respectively, there has been an increasing focus on mechanism and optimization. In line with this trend, AI is especially useful in the study of catalytic cycles and in the development of strategies aiming at improving catalyst performance. Figure 2 presents an overview of key concepts in the application of AI to homogeneous catalysis with TMCs. Circular AI workflows (Figure 2a) can achieve excellent results since they keep improving as new data is acquired iteratively. However, this is often challenging for TMCs considering the type of data that AI needs to be optimized: abundant, complex, and precise.^45^ High-throughput experimentation (HTE) and high-performance computing (HPC), currently propelled by integrated AI-robotics and GPU technologies, respectively, are both important tools to address this problem.

**Figure 2:**
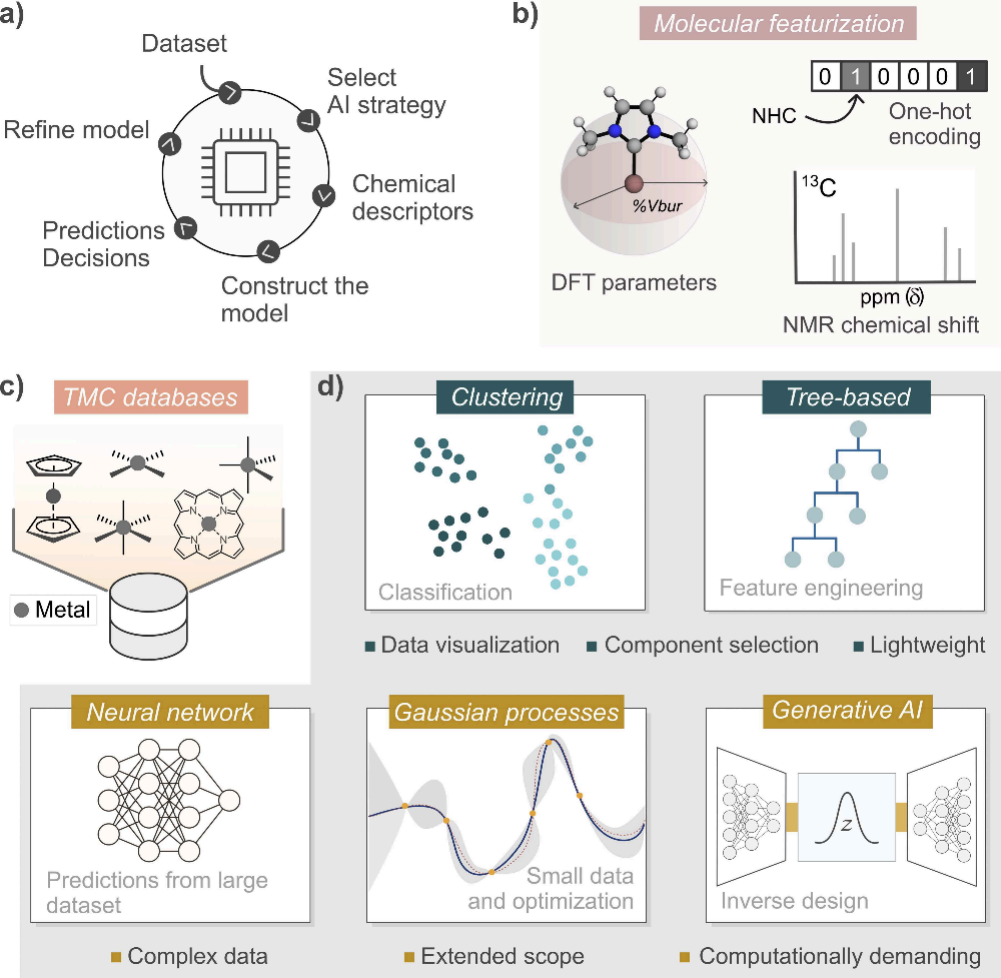
AI framework for homogeneous catalysis with TMCs. (a) Circular, iterative AI workflow. (b) Molecular featurization techniques. (c) TMC datasets with diverse ligands and coordination environments. (d) ML algorithms commonly used in AI for different purposes. TMC = transition metal complex.

Another challenge is how to represent TMCs in AI models in a way that reflects how the intrinsic complexity of the organic ligands is augmented by the inorganic nature of the metal centers. In this regard, several representations have been derived (Figure 2b),^46–49^ using and combining descriptors derived from DFT calculations,^50–53^ structural fingerprints and other chemoinformatics representations available from software like RDKit,^54^ and other features that can be computational, experimental, or hybrid like, for example, NMR signatures.^55,56^ Chemical reaction representations are also generally important in ML for chemistry,^57–59^ though their application to homogeneous catalysis remains seldom explored.^60^ Notable efforts to bridge this gap include the smooth overlap of atomic position (SOAP) descriptor^61^ and the work of von Lilienfeld and co-workers,^62,63^ who developed specialized 3D descriptors to better represent chemical complexes in ML applications.

Datasets are also a key element^64^ since, in general, the quality of the results yielded by AI models is limited by that of the data used to optimize them. Relevant examples include the molecular dataset within the Materials Project^65^ and the Open Catalyst dataset for heterogeneous reactions,^66^ among others.^67,68^ However, the datasets available for TMCs have been smaller in number and size than those available for organic compounds,^69^ though recent efforts,^70–72^ like the tmQM datasets^53,73^ and the associated ligand libraries,^74^ are mitigating this issue (Figure 2c).

Since AI themselves are statistical models, the field has benefited enormously from the research done on ML during the long AI summer that we are still enjoying. Several choices are available, each serving different purposes depending on the data framework (Figure 2d). Clustering^75^ finds patterns in complex data that can be used for classification tasks; for example: active/nonactive catalyst. Methods based on decision trees^76^ are ideal for tabular data and can be used to simplify representations, including only the most important descriptors. Deep neural networks^77^ (DNNs) perform well with large data, making accurate predictions. In small data scenarios, Gaussian processes^78^ (GPs) can perform better than NNs, enabling also optimization tasks based on uncertainty quantification. Finally, generative AI allows for reversing predictive tasks into inverse design,^79^ so that a catalyst is predicted from a user-defined target property instead of the other way around. Together with datasets and representations, these methods enable AI applications to catalysis that can focus on either the ligands^80^ or the whole complexes,^81^ from a perspective based on either the mechanism^82^ or on performance metrics like the reaction yield.^83^

In essence, AI models try to represent a function that maps catalysis descriptors to a figure of merit (for example, yield or selectivity), enabling its prediction. These models need to be *learned* from a body of *training data*; that is, they are optimized to minimize the deviation between what they predict and what is assumed to be true from the data available. The present Perspective discusses this approach, referred to as predictive (or, also, discriminative) AI, together with others that focus on optimization and inverse design. In the broader context of AI, other technologies, including natural language processing^84^ and computer vision,^85^ will be also mentioned. Many of these advances are rooted in computational approaches, underscoring the need for synergizing theory with experiment.

## Mechanism-Based Predictive ML

The study of reaction mechanisms in homogeneous catalysis has been a standard practice in the rationalization of catalytic performance.^86^ Mechanistic investigations provide key insight, such as the resting state, rate-determining step (RDS), and energy span,^87,88^ which contribute to rationalize the activity of the catalyst and other fundamental properties, including robustness^89^ and selectivity.^90^ Conventional approaches focus on the analysis of one or a few metal complexes catalyzing specific reactions, which typically involve fewer than ten consecutive elementary steps in their minimum energy path.^91^ Thanks to these studies, there is extensive information on the mechanisms of the most popular catalytic reactions. For instance, in metal-catalyzed cross-couplings, three main steps are identified: (i) oxidative addition, (ii) transmetalation, and (iii) reductive elimination, with the oxidative addition generally recognized as the RDS.^92^ The use of AI technologies and, in particular, ML, has been envisioned as a new strategy to obtain mechanistic information with reduced computational expense. Current models have proven their ability to predict structure and reactivity properties of transition states (TSs), as well as both in-cycle and off-cycle intermediates,^93^ at a cost significantly lower than that associated with DFT calculations.

### Molecular Volcano Plots

One of the main tools for the computational evaluation of a large number of catalysts for a given homogeneous reaction is the *molecular volcano plot* concept implemented by Corminboeuf and co-workers,^94,95^ which is similar to that developed by Nørskov, based on the application of the Sabatier’s principle to heterogeneous catalysts.^96–98^ In principle, two fundamental assumptions are made when using this tool: (1) the reaction mechanism is invariant to the composition and structure of the catalyst within the space explored, and (2) the chemical system is under thermodynamic control. Regarding the first assumption, potential deviations from the expected reaction pathways should not be overlooked,^99^ and, regarding the second assumption, it should be noted that recent developments allow for tackling more complex scenarios by means of microkinetic modeling.^100^ The volcano plot approach aims at finding linear relationships between a reaction intermediate descriptor and the potential determining step (PDS), where the descriptor is the energy of an intermediate relative to the catalyst resting state, and the PDS is the energy of the most demanding step relative to the descriptor intermediate. When the catalyst candidates are mapped over these two-dimensional space, they form a volcano plot in which the top-performing catalyst candidates appear at the top (Figure 3).

**Figure 3:**
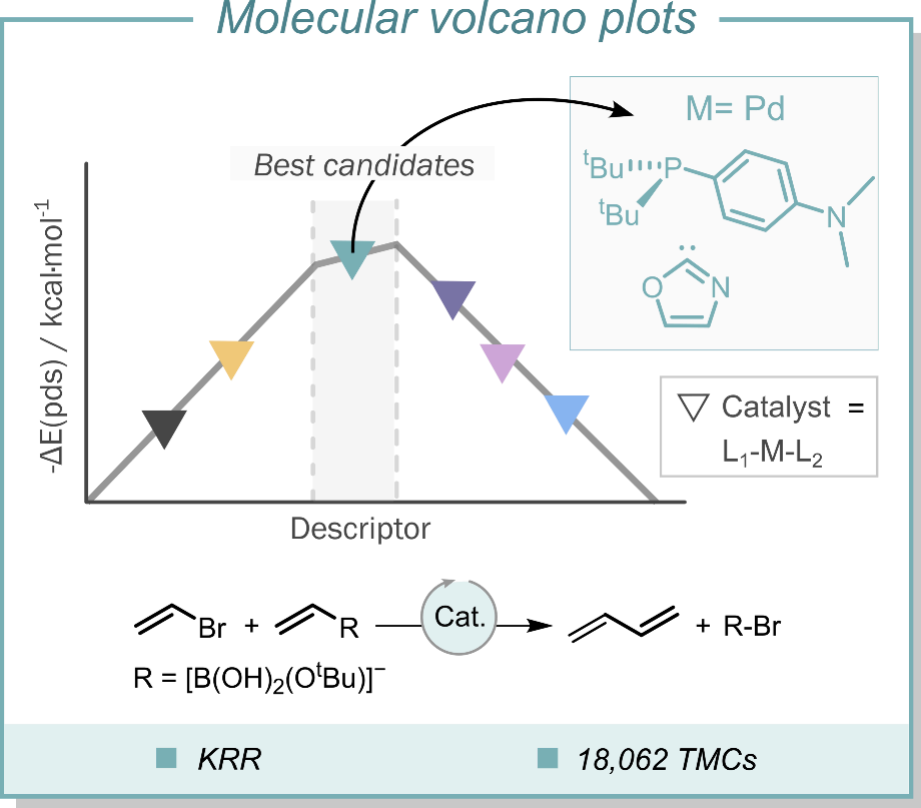
Molecular volcano plot for the Suzuki–Miyaura C–C cross-coupling reaction. Optimal catalysts candidates fall within the gray-shaded region. KRR = kernel ridge regression; TMC = transition metal complex.

In collaboration with von Lilienfeld, Corminboeuf and co-workers combined ML models with linear regression-derived data to define a dense molecular volcano plot of the Suzuki–Miyaura cross-coupling reaction.^101^ This work built on a previous study on a set of 36 catalysts^102^ that was extended to a much larger pool of 18,062 candidates for which 7054 intermediate descriptors were computed. The regressions were fitted with the kernel ridge regression (KRR) method, which has been used extensively in other chemical problems that require finding patterns within nonlinearly correlated data.^103–105^ The KRR models were trained with different molecular representations of electrostatic interactions between atom pairs, including the Coulomb matrix^106^ and the bag of bonds,^107^ along with long-range dispersion interactions.^63^ These representations are part of the quantum-based ML approach^62^ and they can be easily computed with the open-access *QML* toolkit.^108^

The resulting model revealed the presence of 557 systems on top of the volcano plot plateau, thereby facilitating the identification of novel and promising *in silico* catalysts, including also expected moieties, already known to be active, like palladium metal centers supported by phosphine ligands (Figure 3). Furthermore, the identification of the hits was combined with estimated market prices to determine the most cost-effective catalyst candidates. Interestingly, the authors have extended the use of price-based decision-making to other studies, highlighting this pivotal factor in the application of data-driven catalyst discovery to real world problems in industry and academia.^109^ Further synergies between ML and molecular volcano plots have been explored by the Corminboeuf group, including applications to other cross-coupling reactions,^110^ the nickel-catalyzed C­(sp^2^)–O cleavage of aryl ethers,^111^ and the hydroformilation of ethene,^112^ leveraging also unsupervised ML methods, like t-SNE maps.

### Energy Barriers

Besides thermodynamic approaches, catalyst activity can be directly related to reaction kinetics through the computation of the associated TSs which define the critical energy barrier of the reaction mechanism. TS-informed ML models thus incorporate rich information that is highly valuable for any reaction in which the TS determining the performance of the catalyst is well-defined. This approach is also particularly advantageous for stereoselective reactions under Curtin–Hammett equilibrium conditions, where the TS isomers of one concrete step dictate the outcome of the reaction.^113,114^ The main drawback of TS-based models is the need for finding the TSs on the potential energy surface, which is hampered by their complex topology (first-order saddle points) and high computational cost (expensive Hessian matrices). Further, the complexity of TSs from the perspective of electronic structure theory makes their accurate computation more challenging, potentially adding noise to the data needed to train the ML models. In this regard, the difficulty of finding TSs is more pronounced for metal complex catalysts^115^ than for organocatalysts, where the computation of the TSs can be more easily automated by means of SMILES templates.^116^ The intrinsic complexity of the metal–ligand bonds hampers this approach, though promising results have been obtained with force fields fitted for the description of these compounds,^117,118^ which can be also applied to the exploration of extensive reaction networks.^119,120^ Due to these demanding requirements and challenges, examples of TS-informed models are relatively scarce in the literature.^113,121,122^ Here we highlight two studies in which barriers were predicted with distinct representations of the metal catalysts and the associated TSs.

With the aim of mitigating the laborious and expensive calculation of energy barriers, researchers have been investigating their relationships to other molecular properties that, ideally, are both accessible and cheaper. One strategy is to use quantitative structure–activity relationship (QSAR) models, a relatively old concept now re-emerging, propelled by the constant development of ML methods, which have always been at their core.^123^ Following this approach, Friederich and co-workers developed a set of ML models predicting the energy barrier of the oxidative addition of H_2_ to iridium complexes in the chemical space surrounding Vaska’s complex (Figure 4).^124^ In this study, a dataset of 2574 TS geometries, each corresponding to a different metal complex, were formulated and labeled with the associated electronic energy barriers and the distances of the breaking H···H bonds in the TS structures.

**Figure 4:**
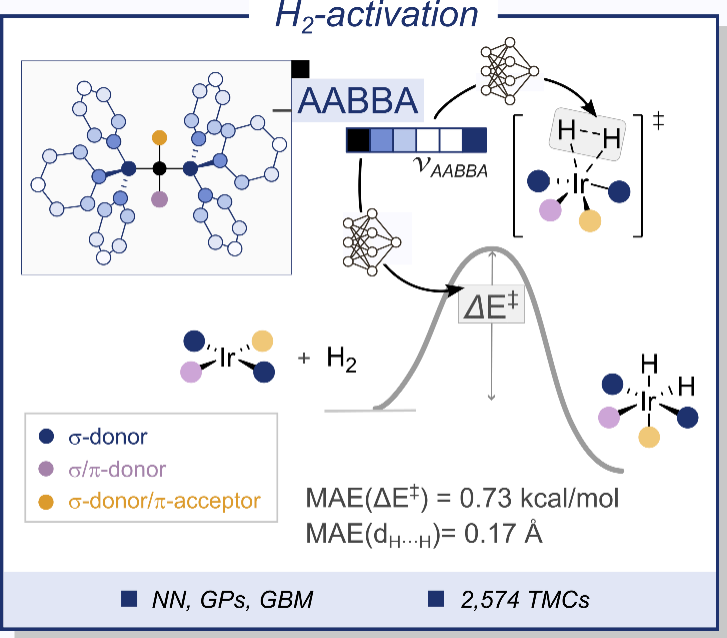
ML approach to the prediction of TS energy barriers and breaking bond distances from autocorrelation vectors on graphs, here applied to the oxidative addition of H_2_ to Ir­(I) complexes in the chemical space surrounding Vaska’s complex. NN = neural network, GPs = Gaussian processes, GBM = gradient boosting machine.

Two distinct ML models were trained with this data to predict the oxidative addition barriers: neural networks (NN) and GPs. The NN models were based on autocorrelation (AC) vectors, which were computed from the molecular graphs representing the metal complexes using the *molSimplify* program developed by Kulik and co-workers.^125^ The hyperparameters of the NNs had a significant impact on the quality of the final model and were optimized using a Bayesian approach.^126^ NNs were also used to predict the TS H···H distances with the aim of recovering the DFT calculations that failed to provide the data needed to train the models. This shows how different ML methods can be used not only to build a predictive model but also to optimize its architecture and extend the training data. The GP models were trained with a mix of ACs features and Morgan fingerprints,^127^ yielding the highest accuracies and the capacity of learning with small data.

Gradient boosting machines (GBMs) were used in both models, albeit for different purposes. With the NN representations, GBMs were used to estimate and rank the importance of the AC descriptors in the predictions made by the model, whereas with the GP representations, GBMs were used for feature engineering, reducing the dimensionality of the input vectors passed to the models. Besides these uses, the GBMs enabled a deeper understanding of the key factors affecting the H_2_ oxidative addition barriers. Once the features were ranked by importance, it was realized that whereas some of them yielded predicted barriers well below the average, others yielded the highest barriers. Of these features, some were Morgan fingerprints encoding molecular fragments like, for example, cyanide and fluoride ligands yielding, on average, low and high energy barriers, respectively. Further natural bond orbital analysis on selected examples allowed for a deeper understanding of these observations on the basis of electronic effects. As depicted in Figure 5, GBMs and, in general, ensemble ML methods based on decision trees, are highly versatile, enabling (1) chemical interpretation based on feature importance, which can also be used for (2) dimensionality reduction, naturally mitigating issues in ML models caused by the so-call curse of dimensionality problem, and (3) predictive models for both classification and regression tasks, often outperforming other, more popular approaches.^128^ Bayesian ridge regression is another approach to feature selection that has proven to be effective in the prediction of enantioselecivity.^129^

**Figure 5:**
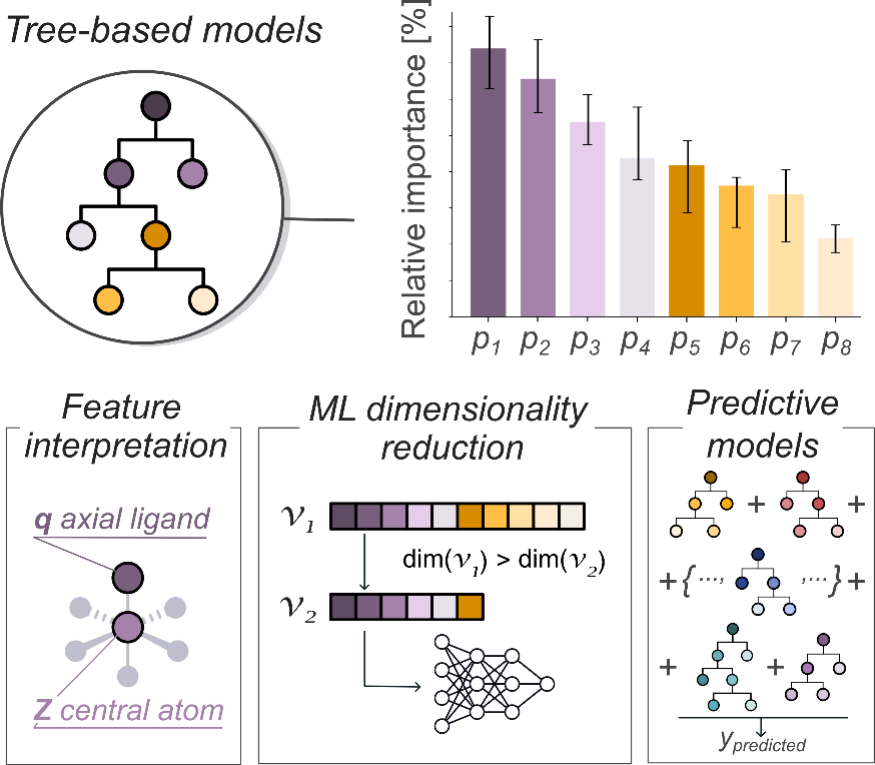
Schematic workflow for ranking features by importance with methods based on decision trees (top) and its various applications (bottom). *Z* = atomic number; *q* = atomic charge.

On a subsequent study, the same Vaska’s complex dataset was used to reassess the performance of the ML models after reformulating the AC representation with the atom–atom bond–bond bond–atom (AABBA) approach.^130^ The AABBA autocorrelations extended the attribution of the molecular graphs by adding bond features to the edges, which are self- and cross-coupled to the atomic features, improving the accuracy of ML models in the prediction of the oxidative addition TS barrier and H···H distance. The definition of the AABBA ACs, which is modular and flexible, depending on various user-defined parameters, was adjusted in systematic experiments in which the dimensionality of the resulting fingerprint vectors was gradually increased. Feature importance approaches based on GBMs were also explored and leveraged in NN and GP models.

TS-informed ML is also a powerful approach to catalytic asymmetric reactions in which the energy difference between diastereomeric TSs in the selectivity-determining step (ΔΔ*G* ^⧧^) defines the enantiomeric excess of the reaction.^131–133^ Hong and Ackermann explored the features approaching a quantitative description of the TSs for a model predicting ΔΔ*G* ^⧧^ in palladium-catalyzed C–H activation asymmetric reactions.^134^ In order to construct a TS-like geometry, the authors employed structural templates for each of the two reaction steps involved in the C–H activation mechanism,^135^ in concert with a subgraph recognition procedure (Figure 6). The templates were designed to reflect the spatial arrangement of the substrates relative to the catalyst, which was determined by precomputing well-defined TSs. In principle, this TS-template concept can be transferred to any metal-catalyzed reaction for which the reaction mechanism is known to have a well-defined TS structure that is not very sensitive to the modification of the catalyst or substrate. The templates were used to extract features related to the TS, including atomic and bond properties, physical organic descriptors, and chirality information, yielding a 29-dimensionality vector that was used as the representation. A regression ML model based on the Extra Trees (Extremely Randomized Trees) algorithm provided the highest accuracy from a pool of eight different predictive models, using 127 data points for training and 28 for testing. This model was further improved with a Δ-ML approach^136^ learning corrections from a baseline, allowing to predict the enantiomeric excess over a large chemical space comprising 846,720 systems.

**Figure 6:**
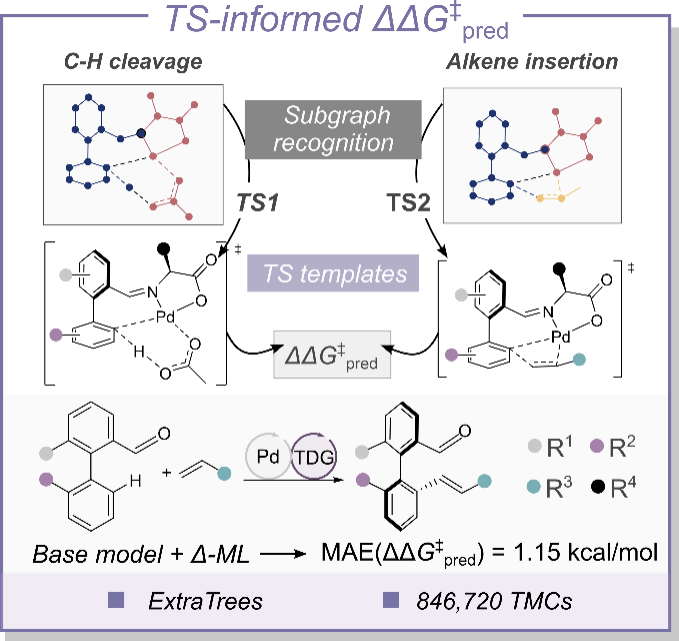
Subgraph recognition-based strategy of the TS structures involved in the C–H activation and the scheme of the asymmetric palladium-catalyzed electro-oxidative reaction. TS1 and TS2 denote TS templates 1 and 2; Pd = palladium; TDG = transient directing group.

Another advantage of this TS-template ML approach is that it provided importance scores for the features used in the predictions, which facilitated the investigation of the origin of the enantioselectivity. In particular, the ML model uncovered molecular interactions that have been overlooked in DFT studies on reference systems. This highlights the importance of synergizing ML and DFT approaches in a feedback loop in which the interpretation of ML models assisted by DFT calculations expands the chemical understanding of complex catalytic reactions. Other successful examples of this approach include applications to the prediction of secondary-sphere effects in organocatalysis,^137^ the solubility of inorganic ion pairs,^138^ and the identification of key off-cycle species in metal-catalyzed reactions.^93,139^

In summary, mechanism-informed ML models can be a powerful approach to catalyst design and discovery though they are intrinsically limited by the need for knowing the reaction mechanism. Further, the mechanism can be subject to complex dependencies leading to unexpected changes in the nature of the preferred reaction pathway, thereby compromising the transferability of the models. Also worth noting are the limitations of the TS-based approach due to the difficulty of finding these stationary points in the potential energy surface. A possible strategy mitigating both these issues would be to integrate automated pathway searchers into the ML pipeline,^120,140^ though these would quickly add to the overall computational cost.

## Mechanism-Agnostic Predictive ML

ML models for catalysis have been also formulated without using information on the underlying reaction mechanism. Instead, the representations provided as input are derived from simple and cheap chemoinformatics data, such as one-hot encoded features or binary fingerprints,^141^ which can also be combined with DFT descriptors.^142^ In these models, it is common practice to combine computationally derived features as input with experimental measurements that are set as the target to predict. Though this approach streamlines the overall workflow, reducing computational costs significantly, it should be used with care, since it may overlook critical factors associated with the reaction mechanisms. In the context of homogeneous catalysis with metal complexes, the models are often used for regression tasks predicting product yields and selectivity.

For yield prediction tasks, the reaction conditions are usually inputted to the model as machine-readable objects encoding various parameters,^83^ including chemical additives, bases, ligands, and reactants, among others. Some studies have also explored the incorporation of experimental data into the input representation. For example, Amir and co-workers developed predictive models using representations informed with NMR spectral data to predict the yield of ester hydrogenation reactions catalyzed by ruthenium.^143^ The accuracy of the yields predicted was very sensitive to the choice of the ML method, with GPs outperforming NN, random forest, and k-nearest neighbors models.

For the prediction of selectivity, mechanism-agnostic models that do not leverage concrete information on the TS or other key elements of the reaction pathway need to be somehow compensated with other meaningful data. This data often consists on descriptors providing a quantitative measure of the electronic and steric properties of the ligands supporting the metal center and the substrates reacting with it. In this regard, feature engineering can be critical in applications to asymmetric reactions,^144^ where it is important to reflect the steric properties of the ligands with descriptors like Sterimol and V_bur_,^145^ which should also be considered for the reactants when predicting regioselectivity.^114,146^ Combined with ML methods and other data-driven approaches, these descriptors have enabled the prediction of the enantiomeric excess for various reactions.^52,128^ Relevant concrete examples include the discovery of highly selective asymmetric catalysts based on the average steric occupancy developed by Zahrt and co-workers,^147^ and the use of mixed electronic and steric descriptors in the discovery of organophosphorous ligands for cross-coupling reactions by Gensch and co-workers.^70^

An intrinsic limitation of these approaches is that the models cannot be directly interpreted from a reaction mechanism perspective, though based on domain knowledge, it could still be possible to relate the latter to appropriate input features or the targets predicted. Further, it is also possible to build ML models that are not mechanism-informed at the input level and yet predict the mechanism itself from experimental observations on the reaction outcome, like, for example, the time-evolution of the concentrations.^148^

## Catalysis Optimization with Active Learning

Besides making predictions for catalysis, one can aim at optimizing the catalytic process itself, thus following an alternative approach that can also be driven by data and ML methods. When optimizing reaction conditions, the number of parameters to consider includes substrate, catalyst, reagents, additives, solvent, concentrations, temperature, and more. All these parameters can have a significant impact on the reaction outcome, making their evaluation critical. The Design of Experiments (DOE) concept^149,150^ was defined as a statistical strategy to identify optimal conditions based on the systematic monitoring of a given reaction. In this regard, the development of advanced high-throughput experimentation instruments,^151^ now combining robotics with AI,^43,44^ has enabled the exploration of large reaction-condition spaces. However, human bias and intrinsic limitations of the experimental setups hamper the exploration of the large chemical spaces resulting from molecular fragment combinatorics.^152^ In practice, reaction yields are usually evaluated for a set of predefined reaction conditions applied to various substrates.^153^ For selectivity, studies often focus on the impact that the structure of the ligands have on the reaction outcome.^154^ From the perspective of transition metal chemistry, the combination of different variables, including not only the ligands but also the coordination geometry, the spin multiplicity, and the oxidation state, yields vast spaces containing from thousands to millions of TMCs.^155^ Adding to this complexity, catalysis is also a challenging multiobjective optimization problem when aiming at real-world applications, in which not only activity, but also selectivity and robustness need to be optimized.

In this context, Bayesian optimization (BO) can be employed to find solutions x* that optimize a complex objective function *f*(*x*) over a design space X :

1 $$x^{*}=\underset{x\in X}{\arg\mathrm{opt}}f\left(x\right)$$

where X can be a chemical space containing catalyst candidates and opt is either a minimization or maximization task that can be implemented with an iterative algorithm aiming at convergence.^153,156–159^ In recent studies, BO strategies utilize ML methods to define *f*(*x*) as a surrogate model that yields the target to be optimized (for example, catalytic activity) together with the uncertainty associated with its computation.^160–162^ The ML method selected for this approach is often a GP, since it intrinsically implements uncertainty evaluation. This is more challenging with NNs, requiring measures within the internal representation (latent space) learned by the model,^163^ though this approach can very efficient when a large and complex dataset is available for training.^164^

Predictive ML is often based on *passive learning* strategies in which the models are trained with large datasets that are fully labeled with the target properties. Though AI methods like deep learning can achieve excellent results with this strategy, there are also relevant caveats. This kind of data is not always available and making it can be very expensive. Further, in multiobjective problems, Pareto fronts, that is, regions of the property space in which two or more targets become jointly optimal, can be largely underrepresented. In these cases, BO methods can be leveraged to implement the opposite strategy: *active learning* (AL).^165–167^ In AL, the training dataset is not labeled and can be regarded as an hypothetical space that is assumed to contain optimal solutions to a given optimization problem. AL starts with a small number of labeled data points and optimizes the *f*(*x*) surrogate model using an acquisition function (AF)^168^ that guides the labeling of additional data points, which are used to retrain the model in an iterative manner (Figure 7). Essentially, the model is recurrently informed with new data points that, in the previous iteration, were predicted to be closer to the target, though with the high uncertainty, in a process that is stopped when either convergence is achieved or enough optimal have been found. As it makes progress toward the Pareto front, the BO method tries to both exploit and explore the space to find more optimal and diverse solutions, respectively. Whereas exploitation can be prioritized with the probability of improvement (PI) algorithm,^168^ exploration can be reinforced by using Thompson sampling.^169,170^ Alternatively, both approaches can be balanced with the expected improvement (EI) approach.^171^ Besides catalysis, but also relevant to it, when dynamics effects are important,^30^ this approach also forms the basis to develop machine-learned interatomic potentials (MLIPs).^172–175^

**Figure 7:**
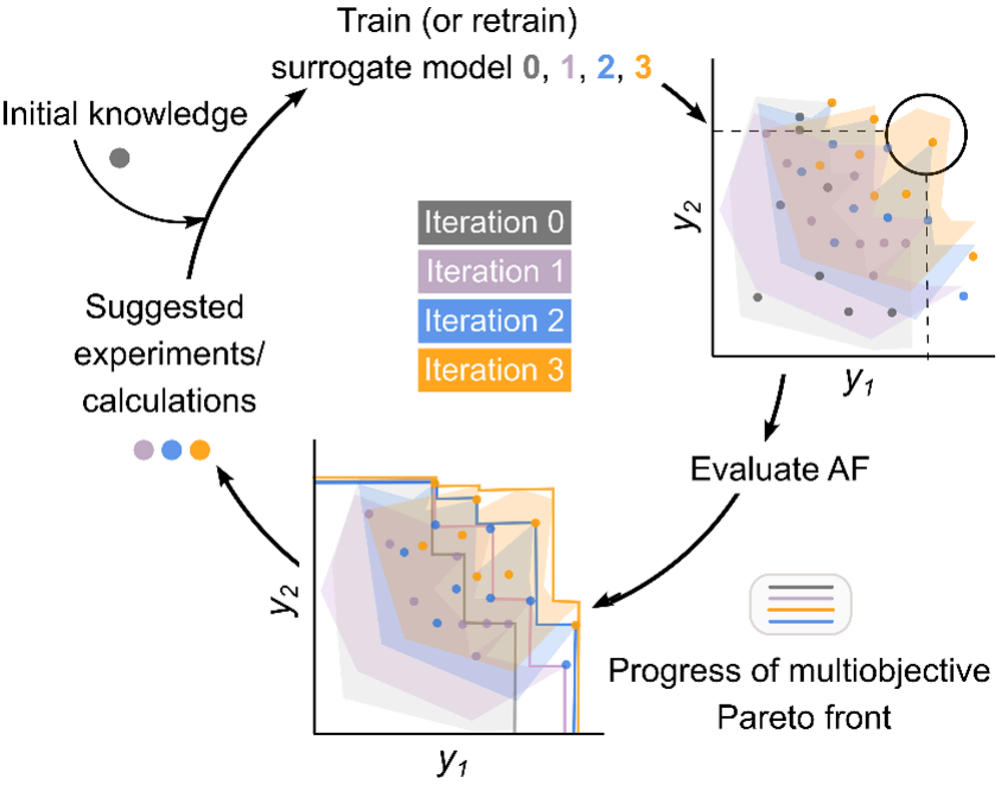
Schematic representation of multiobjective optimization over the Pareto front formed by targets *y*_1_ and *y*_2_ with active learning based on Bayesian optimization. AF = acquisition function.

For homogeneous catalysis with metal complexes, BO-based ML is a powerful approach to the exploration of chemical spaces encompassing millions of catalyst candidates. However, the implementation is challenged by the intrinsic complexity of catalysis, which makes it difficult to formulate the task as a multiobjective problem and, therefore, successful examples remain scarce.^176–178^ We hereby highlight two BO applications: one focusing on the catalyst and the other on the reaction conditions.

### Optimizing Catalysts

Kulik and co-workers have reported several examples of AL optimization applied to TMCs.^155,176,178^ We hereby focus on the optimization of catalysts for the conversion of methane to methanol (Figure 8).^176^ This study is based on a predefined design template consisting of an octahedral metal center with an equatorial tetradentate macrocycle and two monodentate axial ligands, one of them acting as the oxyl radical that reacts over the rebound mechanism.^179^ The metal is either manganese or iron, which can be in different electronic states of low, intermediate, or high spin. The associated combinatorial explosion yielded a search space of 1.2 million catalyst candidates, which upon functionalization of the ligands was extended to a total of 16 million candidates. The functionalization of the macrocyclic ligand was done along the Hammett scale, thus adding both electron-donating and electron-withdrawing groups.

**Figure 8:**
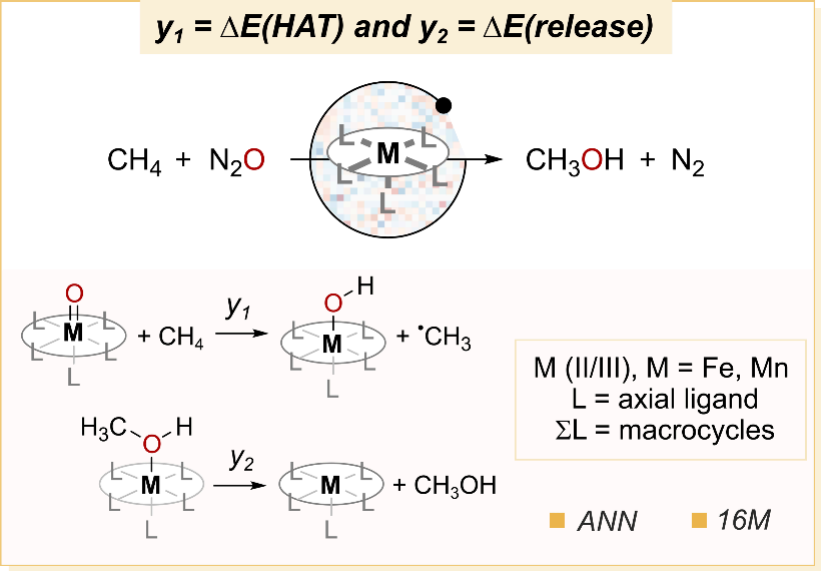
(top) Catalytic oxidation of methane to methanol. (bottom) Reaction steps for which the catalyst was optimized: hydrogen atom transfer (*y*_1_ = Δ*E*(HAT)) and methanol release (*y*_2_ = Δ*E*(release)), both belonging to the radical rebound mechanism.

Two reactions were considered as the key steps of the mechanism: the hydrogen atom transfer and the release of the methanol product (Figure 8), with energies Δ*E*(HAT) and Δ*E*(release), respectively.^176^ Since both processes play a critical role in the outcome of the reaction while being mutually exclusive, the joint minimization of their energies over the Pareto front was used to formulate the catalyst optimization problem. Whereas these energies were gradually computed with a DFT method for an increasing number of metal complexes, as dictated by the AL algorithm, all 16 million complexes were represented by transforming their molecular graphs into revised autocorrelation vectors (RACs).^180^ The surrogate model encoding the trade-off between Δ*E*(HAT) and Δ*E*(release) from the RACs was an NN, whereas data acquisition was guided by a two-dimensional EI function. The uncertainty of the model was quantified in each iteration with a metric based on two components:^163^ (1) the distance between training and test data points once embedded into the latent space of the NN, and (2) a Gaussian distribution that is fitted to normalize and express the confidence of the model. Using this approach, a series of AL searches defined as efficient global optimization (EGO) were conducted to identify high-performance catalysts. Starting from a small subset, 10,000 metal complex hits were selected in each of three generations, which were thereafter filtered down to only 400 by means of *K*-medoid clustering. The analysis of the Pareto solutions found by the algorithm showed that the metal complexes with negatively charged axial ligands were the most efficient at facilitating methanol release while keeping the HAT reaction favorable. Detailed reaction pathway computations on the whole mechanism confirmed that the HAT TS and methanol release intermediate were those determining the catalytic turnover frequency, further supporting the interest of testing the top hits in the wet lab.

Besides favoring exploitation over exploration, or vice versa, the AL method reported by Kulik and co-workers allowed to guide the optimization with a balance between these two strategies by setting a cutoff criterion.^176^ This cutoff, which was known beforehand in this work, can be difficult to define in other applications.^181^ An alternative approach is to use a genetic algorithm with masking functions. Using statistics information generated on the fly, Kneiding and co-workers showed that it is possible to do Pareto front optimizations without any previous knowledge on the limits and relative magnitudes of the targets optimized.^74^

### Optimizing Catalytic Conditions

Another context in which BO-based AL is especially useful is that of modern lab automation, in which intelligent high-throughput robotic systems are being combined with AI methods driven by data that is either mined or collected on-the-fly.^43,44^ This approach has been investigated for organic synthesis,^153,182^ and there are also examples in which TMCs play a major role.^156,159,183–185^ Several platforms are now openly available to implement these workflows: EDBO,^156^ Phoenics,^186^ and Gryffin^187^ for single-objective problems and EDBO+^184^ and Chimera^188^ for multiobjective problems.

In 2022, Grzybowski, Burke, and co-workers reported a protocol combining AL with experimental and computational data to optimize the reaction conditions of the palladium-catalyzed cross-coupling reaction shown in Figure 9.^189^ The starting set of coupling reactants were selected with a clustering approach enhanced with similarity measures based on the Tanimoto coefficient, resulting in 11 substrate pairs. A set of 48 reaction conditions was then defined by considering two temperatures, two bases, three solvents and four catalysts, yielding a total of 528 catalytic experiments that were performed with a robotic system to generate the initial training data. The circular fingerprints of the reactants were combined with one-hot encodings of the reaction conditions to form the representations. The ML model was a GP with a NN kernel, which predicted the reaction yield with the associated uncertainty for any pair of substrates under given conditions. Upon evaluating the yield objective for ensembles of 100 reactions, the uncertainty of each substrate-condition combination was used to guide the selection of other, yet to be tested, substrates, filling sparse regions of the substrate space that incorporated valuable data for the further training of the model.

**Figure 9:**
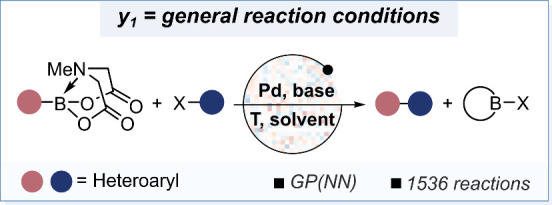
Hetero­(aryl) Suzuki–Miyaura coupling reaction optimized by active learning.

The method implemented and tested in this work provided a novel framework for the assessment of reaction conditions that could be tailored to different substrate combinations, translating into significant savings in time and resources.^189^ This approach might be further augmented in the context of large language models for chemistry,^190–192^ contributing to a major leap in lab automation and autonomous design. However, it is critical to keep sight of a global reaction picture in which, besides successful results, deviations from the expected mechanism or the presence of off-cycle species can produce unexpected results under unexpected conditions. The integration of ML-guided experiments into self-driven laboratories is likely to be still in its infancy and, in line with this, most examples known to date have focused on reactions for which there is plenty of knowledge available. Propelled by the constant growth of research on AI and robotics, including topics like computer vision for the lab,^193^ it is natural to foresee this field expanding toward more and more diverse metal-catalyzed reactions, over a broader range of conditions and techniques for synthesis, characterization, and testing.

Overall, AL can be a robust approach to the data-driven and data-efficient optimization of catalytic processes. However, there are also important limitations to consider. Multiobjective optimizations can be challenged not only by their intrinsic complexity but also by their definition: objectives need to be well-defined, their correlation known, and their acquisition cost reasonable, even in an AL setting if the resources available are limited. The other potential conundrum is how to quantify uncertainty, which is the essential element underlying and driving AL. Gaussian processes and other Bayesian methods are a powerful approach to solving this problem but they rely on functions, like similarity kernels, that can be difficult to define in a sensible manner.

## Generative AI for Inverse Catalyst Design

Molecular discovery with generative AI models is becoming increasingly common in the literature,^79,194–197^ including applications to TMCs.^198–202^ When applied to catalysis, these models are used to go beyond the prediction or optimization of the molecular properties of metal complexes in large datasets, to the generation of chemically valid catalysts that are novel relative to the training data. In terms of output, the fundamental difference between predictive and generative AI is the former returns property values for potential catalysts, whereas the latter returns catalysts that meet a condition, like, for example, being active in a given reaction prompted by a user. This is often termed *in silico* or *de novo* catalyst design,^34,203–205^ which is regarded as one of the “holy grails” in the field.^206^ Generative models are in general more complex and computationally demanding than the predictive, though they also have a key advantage: they bypass the need for explicitly enumerating the entire chemical space of interest which, instead, is formulated and explored implicitly. This is important because even if a predictive model works with computationally cheap features, computing the latter can become extremely expensive when chemists are interested on navigating over millions,^155^ billions,^74^ and even trillions^207^ of compounds.

In this section, we will highlight a few examples based on two distinct generative methods: deep learning, with variational autoencoders (VAE) and diffusion models (DM), and evolutionary learning, with genetic algorithms (GA).

### Deep Learning

Schilter and co-workers developed a VAE for the generation of novel catalysts for the Suzuki-Miyaura cross-coupling reaction.^199^ In general, VAEs are trained to encode the input into a compressed representation in the latent space, which is thereafter decoded back into the same input. Once optimized, the latent space of the VAE can be sampled as a probability distribution to generate new outputs that are different from the inputs forming the training data; here, the inputs and outputs are TMCs, which are represented as graphs.^208^ Further, one can add an auxiliary task in which the latent space representation is used to predict a target property of interest;^209^ in this case, the energy of the first, critical step in the catalytic cycle: the oxidative addition. This allows for biasing the generation of metal complexes toward potential catalysts by minimizing this energy in latent space and then decoding the associated representation into the corresponding TMC. The three components that form the VAE, that is, the encoder, the decoder, and the predictor, are NNs.

The model was trained and tested with a dataset containing 7000 TMCs based on 91 different ligands and six late transition metals, all based in the [ML_2_] formulation.^199^ The generative tasks were conditioned to have the oxidative addition energy within the range from −23 to −32 kcal/mol, as inferred from previous studies based on volcano plots.^101^ These tasks were implemented with a gradient-descent algorithm showing a success rate of 84% in the generation of TMCs that were both chemically valid and novel. Interestingly, a clear move away from known catalysts based on the common phosphine, NHC carbene, and pyridine ligands, showing the capacity of the model to explore underrepresented regions of the chemical space formed by the training dataset. Besides the usual SMILES representation, SELFIES^210^ was also used, showing better performance in terms of the chemical validity of the TMCs generated, which amounted to a total of 8574 novel, potential catalysts. More recently, Strandgaard and co-workers developed a VAE for TMCs that supports the explicit encoding of the metal–ligand bonds, allowing also for two-objective conditioning in generative tasks that can be interpreted and aimed at either the whole metal complexes or the free ligands forming them.^198^

DMs implement a scheme similar to VAEs, in which the encoding is based on a sequential series of noising steps, followed by a decoding process consisting in a reverse denoising process that can also conditioned by target properties. Compared to VAEs, DMs can deliver higher fidelity in the reconstruction of the input and, for 3D objects, including TMCs, they have the advantage of delivering the geometries directly, without the need to process other representations like strings or graphs. As a downside, they are more complex and expensive to train than VAEs. Bhowmik and co-workers reported the OM-Diff model, a DM doing catalyst inverse design for cross-coupling reactions,^201^ based on the same dataset^101^ used to train Schilter’s VAE.^199^ Considering the 3D nature of the input, the model was made equivariant; that is, it respected the nature of both invariant and variant properties relative to the translation, rotation, and reflection of the TMC geometries. OM-Diff allowed for conditioning the generation of the TMCs not only by oxidative addition energies set as targets, but also by chemical composition, since the metal could be chosen among the set {Pd, Pt, Cu}. Jin and Merz reported a similar method, LigandDiff, in which DMs were used to generate ligands that filled in vacant coordination sites in diverse metal centers.^202^

### Evolutionary Learning

GAs evolve solutions that are modular (TMCs are chromosomes) made of blocks (ligands or ligand fragments are gens) that are iteratively changed (mutated) and exchanged (crossed-over) over sequential groups (generations) to optimize a fitness that accounts for catalyst performance (for example, a function depending on reaction energies). AI boundaries can be vague and the question of whether GAs belong to ML has been somehow controversial. Meanwhile GAs can be seen as an optimization method heavily based on heuristics, they also involve several ingredients that are present in ML, including optimization and the use of probability theory and data, which they generate on the fly, in a way similar to AL, allowing also the solution of Pareto front problems.

GAs have been applied to the inverse design of homogeneous catalysts.^211–213^ Notable examples include the evolution of ruthenium metathesis catalysts^214,215^ and molybdenum nitrogen fixation catalysts for the Schrock cycle.^216,217^ TMCs with the formula [Mo­(L)­(L′)_*n*_] (*n* = 1, 2) in trigonal-bipyramidal or octahedral geometries, with L and L′ being anionic and neutral ligands, respectively, were used to formulate a chemical space based on a set of 91 different ligands.^217^ The objective was chosen to be the effective catalysis of the first protonation (Mo–N_2_ + H^+^ → Mo–N_2_H^+^) and reduction (Mo–N_2_H^+^ + e^–^ → Mo–N_2_H) steps in the catalytic cycle. The fitness was composed by combining xTB-computed reaction energies with a synthetic accessibility measure, considering four different conformers for each ligand combination. Additional constraints were imposed on the size of the system (2–100 heavy atoms) and the number of rotatable bonds (20). In total, 1332 and 747 unique TMCs were evolved by the GA for the *n* = 1 and *n* = 2 formulations, respectively. After DFT validation, 96 *n* = 1 and 70 *n* = 2 TMCs remained as potential catalysts, with some of them being further confirmed as such through calculations on their full catalytic cycles.

Besides TMC catalysts, GAs have also been used to find novel homogeneous organocatalysts. A recent example of interest is the Morita–Baylis–Hillman reaction. Here a two step process of lead identification followed by optimization was employed. Lead identification^218^ was performed by searching reaction networks and extracting information about catalytic activity, and in particular, the TS for the rate-limiting step, the proton transfer mediated by methanol. The information achieved at the lead identification step can then be used in the optimization^219^ that used five GA runs of a hundred generations to give 448 unique structures. These were narrowed down biased on synthetic accessibility and rate-determining step barrier, with two systems selected that require only one synthetic step to synthesize. These systems were proven by both DFT and experiment to be more active than the known 1,4-diazabicyclo[2.2.2]­octane (DABCO) organocatalyst (Figure 10). Remarkably, this seems to be the first example ever of an organocatalyst inversely design by an AI model and confirmed in the lab.

**Figure 10:**
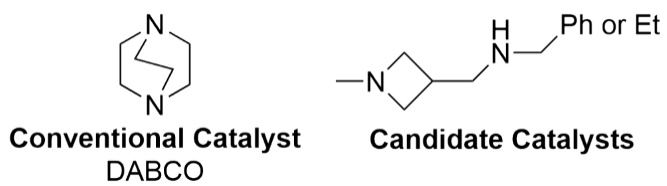
Structure of DABCO, the commonly used organocatalyst for the Morita–Baylis–Hillman reaction, and two improved alternatives identified using a GA.

Another example of organocatalysis discovery is for the Pictet–Spengler condensation carried out using a β-arylethylamine and an aldehyde or ketone.^220^ This case differs in that both turnover frequency (TOF) and enantioselectivity (defined as the ΔΔ*G* ^⧧^ between the two diastereomeric TSs) were simultaneously optimized. The open-source NaviCatGA algorithm^211^ and OSCAR database of organocatalyst building blocks^221^ were used. In order to efficiently calculate these two properties, ΔΔ*G* ^⧧^ was predicted using a statistical model trained on experimental data (820 examples from the literature), whereas the TOF was predicted by a model trained using volcano plots obtained though DFT calculations. The results revealed systems with good performance along the Pareto front. After 32 generations, the median % ee across 50 substrate combinations was 92% ee, up from 86% ee in generation 2, and the median log­(TOF) was 3.3, up from 2.0 in generation 2.

In summary, the change of paradigm from discriminative to generative AI is attractive, giving direct access to the design and discovery of new catalysts. Nonetheless, this approach can also have significant pitfalls. Deep learning methods can be limited by high computational costs, lack of novelty in the generated catalysts, due to bias toward training data, and poor results in multiobjective tasks, which remain challenging and seldom explored. GAs can be limited by low chemical diversity and their tendency to converge properties into local minima. Interestingly, recent research has shown that deep and evolutionary learning methods can synergize,^222^ also at the interface with large language models.^223^

## Unsupervised Learning for Catalyst Discovery

Scenarios in which data cannot be labeled with the target properties of interest are common in catalysis due to its intrinsic cost and complexity. Unsupervised learning methods like clustering then become particularly useful. These methods use statistics and similarity measures on complex data to find patterns that can be leveraged in prediction tasks, though mostly limited to classification.

Schoenebeck and co-workers used this approach to identify ligands that stabilize Pd­(I) dimer catalysts for cross-coupling reactions.^93^ Here an unsupervised *k*-means clustering method^224^ was used, bypassing the need for labeled datasets and experimental data, which are not available for unusual compounds like metal catalyst dimers. In a set of 348 monodentate P-donor ligands from the LKB-P dataset,^225^ those known to form Pd­(I) dimers (P­(^*t*^Bu)_3_, P­(^*t*^Bu)_2_(^*i*^Pr), P­(^*t*^Bu)_2_Ph, and P­(1-Ad)_2_(^*n*^Bu)) were placed in different clusters excluding the PCy_3_ ligand, which was known to not form stable Pd­(I) dimers. The 89 ligands that the algorithm placed in these same clusters were then further studied, generating DFT data for the series of monomeric Pd(0) and Pd­(II) systems as well as dimeric Pd­(I) and Pd­(II) complexes shown in Figure 11. These data were leveraged in a second clustering in which the known Pd­(I) catalyst dimer ligands fell into two clusters, together with 21 other ligands. Synthesis of these species was carried out, leading to the successful synthesis and testing of eight previously unreported catalyst dimers.

**Figure 11:**
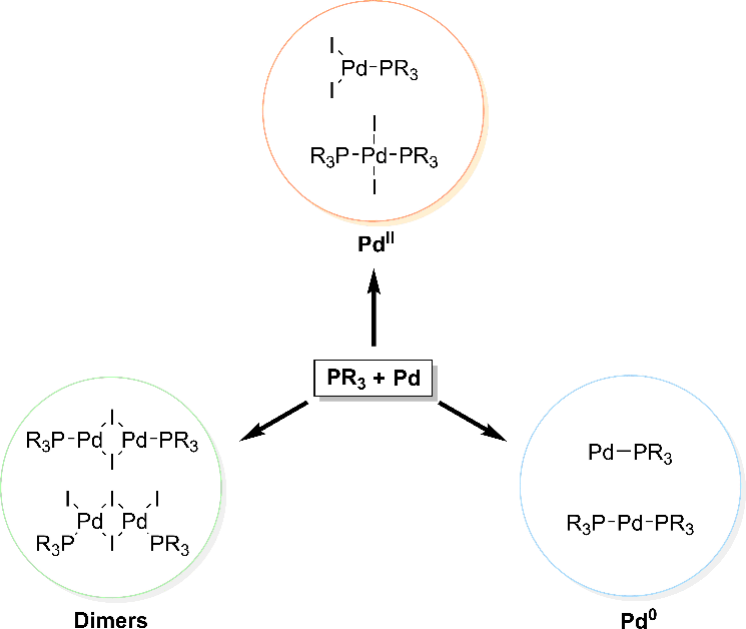
Species labeled with DFT data in the search for stable Pd­(I) dimer catalysts with a *k*-clustering method.

## Conclusions and Outlook

Future development in the application of AI techniques to the field of homogeneous catalysis still faces a few challenges that need to be overcome.^226^ One of them is the need for large, reliable datasets to train and test the models. This is a universal problem that affects other areas of automated, virtual, chemical exploration^227^ but can be a particularly acute problem for homogeneous catalysis with TMCs. Libraries of chemical data generated by computation are becoming increasingly available but are expensive to produce and do not always cover the region of chemical space required for a given problem. Experimental datasets are even more expensive and time-consuming to produce. Defining a method for representing catalysts within a model can also be difficult when a wide variety of approaches are being used.^228^ The best choice can be far from obvious, depending on the combination of several variables, like the ML algorithm, the data available, and the intended task.

In mechanism-informed AI, another challenge is the unforeseen deviation from the expected reaction pathway, which, in the case of TMC catalysts can be easily prompted by open-shell metal centers, spin crossover, and other complex phenomena related to reaction conditions, solvent, and additives. Further, in generative AI, when the chemical space is explored nearby the limits of the training dataset, other mechanisms may begin to operate which could be advantageous, but also detrimental to the targeted catalyst properties. Some of the methods referred in this Perspective may have difficulties in detecting or modeling these effects, which would be important to realize before passing a potential catalyst candidate to experimental testing.

Despite these hurdles, the future for AI and ML in homogeneous catalyst discovery is bright, in a field that is rapidly advancing. Further developments in ML like, for example, foundation models for molecular dynamics simulations, may also trigger and push further advancements. Another area that holds a lot of potential is closer integration with experiment by taking novel, promising hits from models and synthesizing them to verify their catalytic performance, closing a symbiotic feedback loop in which both positive and negative results, reflecting success and failure, will be highly valuable. A step further in this direction would be the full integration of AI into automated laboratories^42,229^ allowing for catalyst discovery based on directional high-throughput screenings. In this context, cointelligent approaches, in which human and artificial intelligences synergize toward a common goal,^230,231^ can also be a fruitful direction.

In summary, AI approaches are being increasingly applied to homogeneous catalysis to gain mechanistic insight, screen large chemical spaces, optimize reactions, and discover novel catalysts. This Perspective has illustrated the current state of the art of the field with selected examples. Although these methods have not been around for long, they are fast becoming widespread and are very likely here to stay and expand. With further work to overcome current hurdles and to improve integration with experiments, in the near future we expect to see AI pushing the understanding and discovery of novel catalysts toward new horizons.
